# FPGA-Based High-Performance Embedded Systems for Adaptive Edge Computing in Cyber-Physical Systems: The ARTICo^3^ Framework

**DOI:** 10.3390/s18061877

**Published:** 2018-06-08

**Authors:** Alfonso Rodríguez, Juan Valverde, Jorge Portilla, Andrés Otero, Teresa Riesgo, Eduardo de la Torre

**Affiliations:** 1 Centro de Electrónica Industrial, Universidad Politécnica de Madrid, José Gutiérrez Abascal 2, 28006 Madrid, Spain; jorge.portilla@upm.es (J.P.); joseandres.otero@upm.es (A.O.); teresa.riesgo@upm.es (T.R.); eduardo.delatorre@upm.es (E.d.l.T.); 2 United Technologies Research Centre (UTRC), Penrose Wharf, Cork T23 XN53, Ireland; valverj@utrc.utc.com

**Keywords:** edge computing, Cyber-Physical Systems, FPGAs, Dynamic and Partial Reconfiguration, energy efficiency, fault tolerance

## Abstract

Cyber-Physical Systems are experiencing a paradigm shift in which processing has been relocated to the distributed sensing layer and is no longer performed in a centralized manner. This approach, usually referred to as Edge Computing, demands the use of hardware platforms that are able to manage the steadily increasing requirements in computing performance, while keeping energy efficiency and the adaptability imposed by the interaction with the physical world. In this context, SRAM-based FPGAs and their inherent run-time reconfigurability, when coupled with smart power management strategies, are a suitable solution. However, they usually fail in user accessibility and ease of development. In this paper, an integrated framework to develop FPGA-based high-performance embedded systems for Edge Computing in Cyber-Physical Systems is presented. This framework provides a hardware-based processing architecture, an automated toolchain, and a runtime to transparently generate and manage reconfigurable systems from high-level system descriptions without additional user intervention. Moreover, it provides users with support for dynamically adapting the available computing resources to switch the working point of the architecture in a solution space defined by computing performance, energy consumption and fault tolerance. Results show that it is indeed possible to explore this solution space at run time and prove that the proposed framework is a competitive alternative to software-based edge computing platforms, being able to provide not only faster solutions, but also higher energy efficiency for computing-intensive algorithms with significant levels of data-level parallelism.

## 1. Introduction

The traditional Wireless Sensor Network (WSN) paradigm, based on a distributed sensing scheme followed by a centralized processing of data, is being shifted by the emergence of edge-centric computing [1,2]. More computational resources are being placed at the edge of data acquisition networks, physically distributing not only the sensors but also the computational resources, with the aim of reducing the communication overheads and the response latency of the network, while increasing scalability and processing performance.

One of the major fields where WSNs are applied nowadays are Cyber-Physical Systems (CPSs), where WSNs act as a bridge connecting the *physical* and the *cyber* worlds [3]. In this regard, let us think about some inspiring scenarios where CPSs are playing an important role. It is not unreasonable to foresee, for instance, a near future with networks of autonomous self-driving cars going around our cities, deeply sensing and understanding the environment, while making run-time decisions about where to go and how to deal with the countless number of potential situations that may appear during each route. Smart grids composed of millions of consumers connected to thousands of energy production centers need to make a great deal of decisions at run-time in order to increase the operation efficiency of generators and distributors, allowing flexible usage choices for consumers, while being able to balance the peak and average demands of electricity. The Industry 4.0 revolution envisages digital factories where production machines are interconnected providing intelligent, resilient and self-adaptable operation, working together in a more cost-effective and efficient manner. In all those scenarios where CPSs are becoming ubiquitous, multiple domain-specific sensor and actuator networks are integrated under collaborative and distributed decision-making schemes, raising the level of autonomy of the systems, but also their complexity and computational demands. These scenarios show how highly constrained networked embedded systems have evolved to cope with the requirements imposed by CPSs, bringing more resources to the edge and conforming the foreseen scenario of seven billion people surrounded by seven trillion devices.

Edge Computing makes necessary to seek alternatives to the use of low-profile microcontrollers as it has been traditionally done in WSNs [4]. When algorithms become more computing intensive, Field Programmable Gate Arrays (FPGAs) can prove beneficial when used as processing platforms [5,6,7]. Moreover, System-on-Programmable Chips (SoPCs), integrating FPGAs with microcontrollers on the same device, allow combining the flexibility of software with the performance of hardware. While some FPGA technologies can provide competitive power consumption rates (e.g., Flash-based), only SRAM-based FPGAs can meet the requirements imposed by high-performance embedded applications. The price to be paid for this performance is power consumption, and therefore, additional power management strategies need to be implemented to maintain the energy efficiency always required in traditional WSNs.

On top of that, some other non-functional requirements for CPS edge computing platforms are dynamic adaptability and dependability. Adaptability is derived from the need of interaction with a changeable physical world, while dependability is related with the criticality of the physical entities controlled by these systems. Whenever these non-functional requirements are present on the Edge, the applicability of other competitor hardware architectures such as domain-specific accelerators or embedded GPUs is reduced. In this regard, the run-time reconfiguration capabilities available in SRAM-based FPGAs, which enable the time-multiplexing of computing resources, make them appropriate for the adaptability levels required by CPSs. However, specific techniques are required to enhance the dependability of solutions running on FPGAs.

In this paper, a framework to design adaptive high-performance embedded systems for edge computing is presented. The framework is based upon three elements: an architecture for flexible hardware acceleration, called ARTICo^3^, an automated toolchain to build FPGA-based reconfigurable systems, and a run-time execution environment to manage running applications.

The ARTICo^3^ architecture offers hardware-based, dynamically-adaptable, high-performance embedded computing, exploiting both task-level and data-level parallelism. The use of Dynamic and Partial Reconfiguration (DPR), together with a slot-based partitioning of the reconfigurable fabric, allows module replication and, in some cases, even relocation. Hence, application-specific hardware accelerators can be instantiated as many times as required (i.e., replicated using hardware “*copy and paste*”), provided that there is enough space available, to better exploit parallelism at both task and data levels. An optimized, bus-based and DMA-powered communication infrastructure is used to feed the accelerators and retrieve data when computing is finished. This infrastructure transparently maps data transfers between an external RAM memory and the hardware accelerators. Moreover, the mapping strategy can be dynamically changed when multiple copies of an accelerator are instantiated in the reconfigurable region. In addition, the architecture features dedicated modules to enhance the functionality of the system, once combined with the adequate mapping strategy. The data delivery pattern that enables redundant execution is complemented by a voter unit that is inserted in between the returning datapath from the accelerators to achieve fault-tolerant execution. Moreover, the mapping strategy that enables parallel execution is complemented with a hardware reduction unit, able to perform atomic operations (addition, maximum, minimum, etc.) on the fly, i.e., during data transfers, and merge data coming from different accelerators without additional overheads that would compromise computing performance.

The use of complex hardware-based processing architectures for high-performance embedded computing has been around for a certain amount of time. However, the lack of both design- and run-time support made it extremely difficult for these platforms to become widely used by the general public. In this regard, the proposed architecture is complemented with a toolchain to automatically generate ARTICo^3^-based systems from high-level descriptions (C/C++ code) of both application and hardware accelerators using High-Level Synthesis (HLS), even though legacy designs using HDL are also supported.

Moreover, to ease the development of user-defined applications using the ARTICo^3^ framework, a runtime library (accessible through a simple API) has been developed. Run-time mechanisms such as FPGA reconfiguration or parallel-processing deployment using DMA transfers are also managed transparently, making it possible for designers without prior knowledge of FPGA-based processing systems to implement fully-fledged DPR-capable systems. Furthermore, the execution model associated with the architecture, mainly based on massive data-level parallelism, provides transparent scalability in terms of computing performance, a fact that can be also combined with energy efficiency or fault tolerance to cover the full spectrum of available solutions when using the ARTICo^3^ architecture.

In summary, the main contributions of this paper are:

- A dynamically reconfigurable architecture for hardware-accelerated edge computing that supports tradeoffs between computing performance, energy consumption and fault tolerance (driven by user applications) at run-time.
- An optimized on-chip communication infrastructure to enable configurable, DMA-powered memory transactions.
- An automated design methodology to generate custom FPGA-based edge computing systems from either high-level (HLS) or RTL descriptions.
- A run-time scalable parallel execution model for FPGAs that exploits data-level parallelism and is based on DPR.

The rest of this paper is organized as follows. Section 2 presents an overview of the related work. The ARTICo^3^ framework is presented in Section 3, and validated through experimental results in two scenarios in Section 4. Finally, Section 5 presents the conclusions and the future work.

## 2. Related Work

For the sake of clarity, this section is divided into two subsections. The first one addresses the importance of FPGA technology in the context of CPSs, whereas the second one presents different FPGA-based embedded processing architectures, design tools, and run-time management techniques.

### 2.1. CPSs and FPGAs

As it was already envisioned in [8], the *cyber* part of CPSs, usually conformed by embedded systems, has an inherent need for networking. Hence, it is common to establish clear relationships between CPSs and the Internet of Things (IoT) paradigm. Moreover, resource limitations in current end devices (i.e., the edge of the network) demand additional computing power either in the cloud or closer to the edge (i.e., fog computing [9,10]).

The use of FPGAs (or even SoCs with FPGA parts) in elaborated IoT scenarios can be seen throughout the literature. For instance, Venuto et al. [11] presented an FPGA-based CPS built around wearable IoT technology that includes complex machine learning algorithms for healthcare applications. The work presented in [12], on the other hand, proves that FPGAs provide better results than optimized software running in GPUs when implementing Binarized Neural Networks [13]. Both examples show a promising path for embedded machine learning in IoT using reconfigurable technology inside edge devices.

The research around FPGAs for IoT spans a wide range of topics, from new FPGA technologies to enable the ultra-low power consumption rates required in some applications on the edge [14], to different processing architectures, either with or without DPR capabilities, to be implemented on top of the FPGA substrate. For instance, Johnson et al. [15] presented a DPR-enabled security-oriented architecture for IoT applications. However, it does not support dynamically scalable execution of hardware accelerators (only one accelerator is assumed per function) as in the ARTICo^3^ architecture. Another architectural example can be found in [16], where a low-end node for resource constrained IoT deployments is presented, but where DPR is not supported due to the FPGA being flash-based.

### 2.2. FPGA-Based Embedded Processing: Architectures and Tools

The use of reconfigurable technology adds new challenges to the already huge design space of multiprocessor systems (this section provides a brief overview on Reconfigurable Computing Architectures and methodologies; please refer to [17] for a more in-depth reading on the matter). These challenges have been divided in the literature in several categories [18]: architectures, tools for design space exploration, simulation and programming, and infrastructures and Operating Systems (OS) for run-time operation.

#### 2.2.1. Architectures

Architectural support for reconfigurable computing has been widely studied and therefore, several examples can be found in the literature. ReCoBus [19], for instance, shows a custom bus-based architecture where reconfigurable modules are dynamically attached to a bus communication interface, as opposed to the approach applied in ARTICo^3^, in which accelerators are not attached to the bus directly but to a configurable gateway to support dynamic datapath changes. ReCoBus allows interconnections between reconfigurable modules by an architecture called I/O bars to achieve point to point communication among accelerators. Although this is an interesting approach, it is different from the proposal of ARTICo^3^, where accelerators do not share data to enable a scalable and data-parallel execution model. These I/O bars allow direct interfacing with on chip memory and I/O resources. All the reconfigurable modules having access to a shared memory can lead to memory hazards that need to be taken care of by a complex scheduler. In ARTICo^3^, each accelerator has a local memory and they have no master capabilities over the bus. ReCoBus allows loading hardware modules with different sizes, as it is done in ARTICo^3^. Although replication to increase performance and redundancy could be potentially added, there is no specific reference to that or how this could be addressed. Another example can be found within GUARD [20], which is a methodology to increase fault tolerance in SRAM-based FPGAs on demand. It includes a generic architecture where reconfigurable modules are attached to a bus. By monitoring error rates and some other parameters, the level of required redundancy is managed. As in ARTICo^3^, the static part is assumed to be hardened by other methods and they are only focused on adding redundancy to the dynamic region.

However, there are additional challenges derived from having reconfigurable hardware architectures that need to be taken into account. The first challenge is related to the number and type of processing elements (PEs) used. The reconfiguration of PEs can be carried out from two different perspectives: virtually adapting the inner datapath of the PE using multiplexers [21], or actually modifying the configuration memory of the FPGA to completely change the PE in a coarse-grain DPR approach, which is the one followed in ARTICo^3^. An example of the first type of reconfiguration can be seen in [22], where the authors use Reconfigurable Instruction Set Processors (RISPs) to change instructions at run time, being able to modify the functionality of the PEs. Alternative techniques such as virtual reconfiguration [23], or multiplexing similar to in [24] can be also used to change the configuration of PEs. This reconfiguration approach can be faster, since less data need to be configured but it is also less flexible, due to the need of having fixed logic resources. When working with coarse-grain DPR (e.g., ReCoBus), on the other hand, each PE can have different memory structures and therefore, the PE is usually reconfigured together with its local memory [25], which is also the case of ARTICo^3^-based accelerators. The use of reconfigurable infrastructures such as the MORPHEUS platform [26], with different levels of reconfiguration granularities for real-time processing, has also been analyzed.

The second challenge for reconfigurable architectures is how to handle communication interfaces among the PEs, since both the number of them and their locations can be changed dynamically. Important aspects are, for example, the architecture reduction on demand, i.e., changing the number of communication ports according to the changing number of PEs. Depending on the number of processors, different approaches are used: point-to-point communications for a low number of PEs, bus-based architectures for medium, and Networks on Chip (NoCs) for a higher number of processors. Star-wheels [27] or CoNoChi [28] are examples of NoCs, or the work presented in [29], where the number of processors can be changed at run time while DPR is used to change the number of switches on demand. In [19,30], it is possible to find bus architectures capable of changing the number of PEs. This is also the approach applied in ARTICo^3^.

The third challenge related to reconfigurable architectures is the memory structure. As it was already mentioned, PEs can have different memory structures and thus, sometimes, it is convenient to reconfigure the whole block. Besides, architectures based on shared memory might need to handle a changeable amount of agents accessing the memory as shown in [31,32]. A particular example of memory structure can be seen in VAPRES [33], where run-time assembly of streaming processors with hardware module replacement and communication reconfiguration is done. In ARTICo^3^, the memory structure is hierarchical and has three different levels: global (shared with host microprocessor), local (common to all logic within an accelerator), and registers (fast access storage within an accelerator).

#### 2.2.2. Design Tools

The complexity faced by developers at design time is a huge concern when working with dynamically and partially reconfigurable systems built on FPGAs. As a result, several toolchains have been introduced throughout the years to increase productivity and make low-level technology dependencies transparent for designers. In this regard, RecoBus-Builder [34] is presented as a means to easily build DPR-enabled systems based on the ReCoBus architecture. It not only generates the communication infrastructure for the reconfigurable partitions, but also provides a ready-to-deploy configuration bitstream to be loaded in the FPGA. The ARTICo^3^ toolchain does not generate the communication infrastructure (it is part of the static region), but does also provide output binaries to program the FPGA and the host microprocessor. Another academic tool is GoAhead [35,36], which goes one step further and enables the floorplanning and automatic generation of DPR-enabled systems with no initial restrictions in the communication infrastructure. The flow also supports relocation of partial bitstreams, i.e., being able to move a certain circuit from one reconfigurable region of the FPGA to a different one. Although relocation capabilities have not been enabled for all target devices, the ARTICo^3^ framework can rely on legacy tools for relocatable bitstream generation [37].

Apart from academic tools, there are also some commercial solutions aimed at system-level design for FPGAs. Recently, Xilinx has introduced the so-called SDx design flows, which include SDAccel and SDSoC. SDAccel enables FPGA-accelerated datacenter development by using HLS from OpenCL code. The ARTICo^3^ and OpenCL execution models are similar in some aspects, since both aim at exploiting massive and explicit data-level parallelism. However, while SDAccel also relies on DPR to load accelerators in the FPGA, the reconfiguration granularity is coarser than the one used in ARTICo^3^, where individual accelerators (and not a full set of them) can be changed. Moreover, SDAccel requires PCIe coprocessing boards for application deployment; thus, it is not a suitable solution for the Edge. SDSoC, on the other hand, targets embedded systems and uses HLS to generate accelerators from C or C++ code. In terms of design automation, SDSoC and the ARTICo^3^ toolchain have some common features such as automatic and transparent system generation with communication infrastructures and HLS-based accelerator design. Nevertheless, the main difference between ARTICo^3^ and both SDAccel and SDSoC is the run-time component of the ARTICo^3^ framework, which allows transparent and scalable data-parallel processing with selectable fault tolerance and energy efficiency using DPR. Table 1 shows the main similarities and differences between the ARTICo^3^ framework and alternative tools. The last column refers to the possibility of performing user-driven tradeoffs between computing performance, energy consumption and fault tolerance at runtime.

#### 2.2.3. Run-Time Support

Dedicated hardware accelerators working as software-like threads can improve computing performance. Moreover, the way they are managed can help cover not only a performance increase but also control over energy consumption and dependability needs. However, even though pure hardware solutions can be optimal, hybrid solutions taking advantage of software flexibility and hardware processing power are shown to be more efficient [38]. The concept of hardware threads is studied in [39] as part of ReconOS, an OS for reconfigurable computing. Hardware threads are treated as additional processes by the OS. Each hardware thread has two interfaces: one with system memory and another with the OS. This approach is similar to the one followed by ARTICo^3^, which provides two interfaces (control and data) and works under an OS. However, in the case of ARTICo^3^, hardware accelerators have no master rights over system memory since those rights can cause conflicts or an additional complexity in the management of the communication protocol. Another example of the use of hardware threads can be found in [40]. The work presents an architecture that allows dynamic allocation of processing units and multithread management at OS level. It includes point to point streaming communication among hardware threads. These streaming interfaces are configurable at run time. In addition, there is a resource manager that uses certain identifiers for the management of the hardware threads, called stubs, as it does with software threads. More examples of OS-level run-time management of reconfigurable systems can be found in [41,42].

## 3. The ARTICo^3^ Framework

The proposed framework provides three components required in high-performance embedded processing solutions: a multithreaded reconfigurable processing architecture, an automated toolchain to deploy edge computing applications using it, and a run-time environment to manage the implemented systems. The ARTICo^3^ architecture is device agnostic. However, the need for low-level DPR support, which is highly technology-dependent, limits the range of devices that can be used as targets. Only Xilinx devices are supported in the current version of ARTICo^3^.

**Figure 1:**
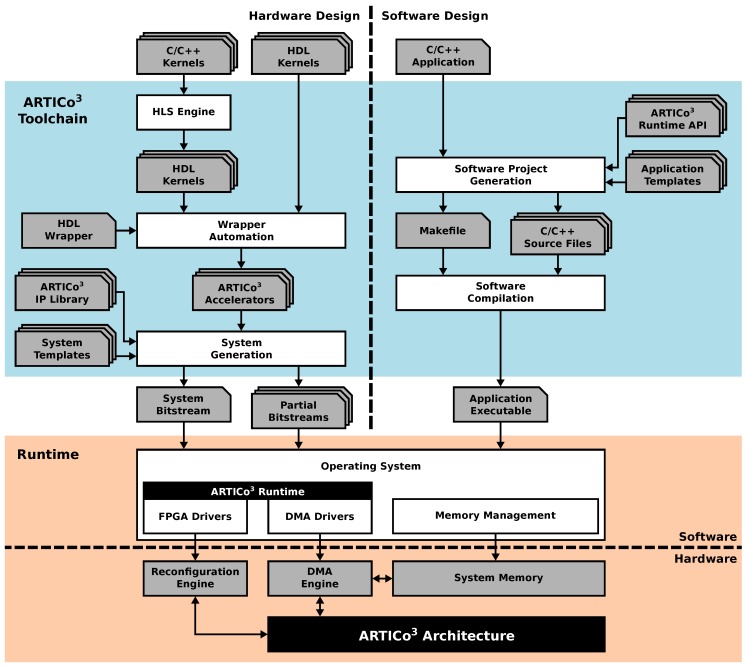
The ARTICo^3^ Framework.

An overview of the ARTICo^3^ framework is presented in Figure 1: all user input files (application definitions) appear at the top; the toolchain that generates both the hardware files to reconfigure the FPGA and the application executable that will run in the target platform (hardware and software components, respectively) appear in the middle; and the final platform, managed by an OS and making use of the ARTICo^3^ architecture through its runtime, appears at the bottom. The following subsections cover each of these components in depth.

### 3.1. Architecture

ARTICo^3^ stands for Reconfigurable Architecture to enable Smart Management of Computing Performance, Energy Consumption, and Dependability (*Arquitectura Reconfigurable para el Tratamiento Inteligente de Cómputo, Consumo y Confiabilidad* in Spanish).

The architecture was originally conceived as a hardware-based alternative for GPU-like embedded computing. Although additional features have been added to this first concept to make it suitable for edge computing in CPSs (e.g., energy-driven execution, fault tolerance, etc.), the underlying hardware structure still resembles GPU devices in the way it exploits parallelism. As a result, for the architecture to be meaningful, applications are required to be a combination of sequential code with data-parallel sections (host code and kernels, respectively). The host code runs in a host processor but, whenever a section with data-level parallelism is reached, the computation is offloaded to the kernels, implemented as hardware accelerators. Moreover, data-independent kernels can be executed concurrently, extending the parallelism to task level.

The key aspect of the ARTICo^3^ architecture is that it offers the possibility of adjusting hardware resources at run-time to comply with changing requirements of energy consumption, computing performance and fault tolerance. Hence, the working point of the architecture can be altered at run-time from the application code, dynamically adapting its resources to tune it to the best tradeoff solution for a given scenario.

As in most DPR-enabled architectures, ARTICo^3^ is divided into two different regions: static, which contains the logic resources that are not modified during normal system execution, and dynamic (or reconfigurable), which contains the logic partitions that can be changed at run-time without interfering with the rest of the system. The reconfigurable region is, in turn, divided into several subregions to host different hardware accelerators. The partitioning of the dynamic region follows a slot-based approach, in which accelerators can occupy one or more slots, but where a given slot can only host logic from one hardware accelerator at a given instant. It is important to highlight that, although the static region is completely independent of the device in which the architecture is implemented, the reconfigurable region has strong, low-level technology dependencies: the size of the FPGA or the heterogeneous distribution of logic resources inside the device impose severe restrictions on the amount and size of the reconfigurable partitions.

The communication infrastructure of ARTICo^3^, optimized for the hierarchical memory approach of the architecture, uses two standard interconnections, one for control purposes (register-based with AXI4-Lite protocol) and another fully dedicated to move data using a burst-capable DMA engine (memory-mapped with AXI4-Full protocol). A gateway module called Data Shuffler acts as a bridge between the twofold bus-based approach of the static region and the reconfigurable hardware accelerators, supporting efficient data transfers between both domains. This bridging functionality is used to mask custom point-to-point (P2P) communication channels behind standard communication interfaces (AXI4 slaves) that can be accessed by any element in the static region (e.g., the host microprocessor for configuration/control purposes, or the master DMA engine for data transfers between external memory and accelerators). This communication infrastructure constrains data transactions between external memory and local accelerator memories to be burst-based and memory-mapped. Application-specific data access patterns can still be implemented, but need to be managed explicitly either in software (the host application prefetches and orders data in the DMA buffers) or in hardware (the user-defined accelerator logic accesses its local memories with the specific pattern, providing additional buffer descriptions as required).

**Figure 2:**
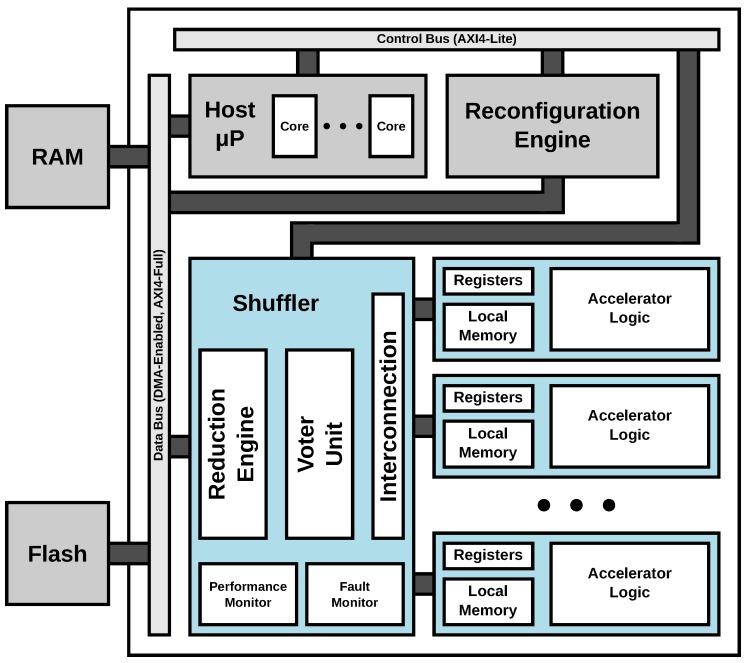
Top-level block diagram of the ARTICo^3^ architecture.

The top-level block diagram of the ARTICo^3^ architecture, with the distribution of both static and dynamic partitions, together with the communication infrastructure, is shown in Figure 2. The ARTICo^3^ framework does not impose where the host microprocessor should be: it can be either a soft core implemented in the static partition of the FPGA, or a hard core tightly-coupled with the SRAM-based FPGA fabric.

**Figure 3:**
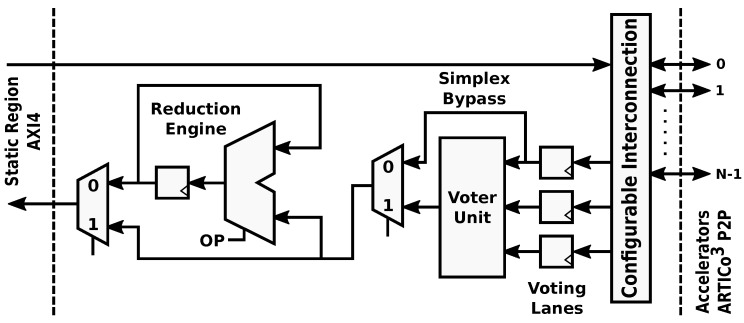
ARTICo^3^ datapath. Its configurable structure allows dynamic changes to support fault-tolerant operation using the voter unit, parallel execution using the Simplex bypass, and data reduction using the configurable reduction engine.

The internal architecture of the Data Shuffler module enables dynamic changes in the datapaths to and from the reconfigurable hardware accelerators. Write and read channels alter their structure to fit a specific processing profile, which is defined by a combination of one or more of the supported transaction modes: parallel mode, redundant mode or reduction mode. Note that the convention used here implies that write operations send data to the accelerators, whereas read operations gather data from the accelerators. The dynamic implementation of the datapath in the Data Shuffler is shown in Figure 3.

The different transaction modes supported by the architecture and enabled by the Data Shuffler can be summarized as follows:

- Parallel mode: Different data to different hardware accelerators, SIMD-like approach (parallel execution, even though the bus-based nature of the communication infrastructure forces all transfers to be serialized between Data Shuffler and external memory). Targets computing performance and energy efficiency.
- Redundant mode: Same data to different hardware accelerators, majority voting for fault-tolerant execution. Targets dependability.
- Reduction mode: Different data to one or more hardware accelerators, accumulator-based engine to perform computations taking advantage of data serialization in the bus. Targets computing performance.

**Figure 4:**
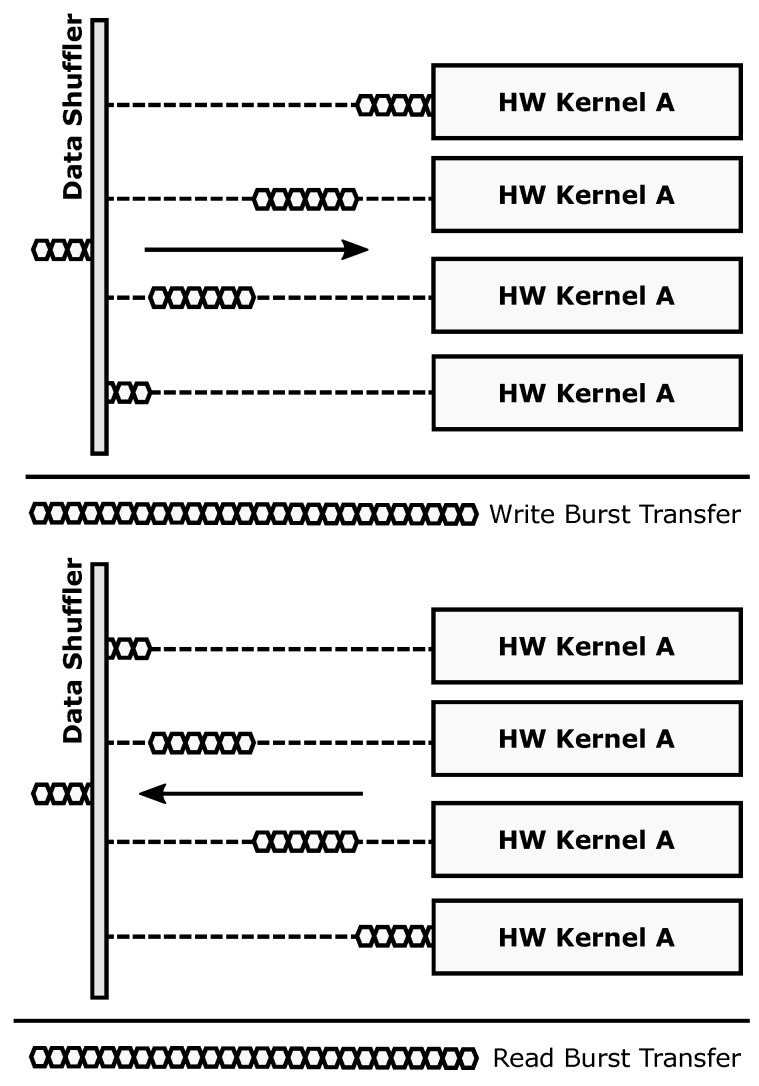
Parallel operation mode.

The parallel transaction mode (Figure 4) is used to take full advantage of data-level parallelism when more than one copy of a given accelerator is present. Burst-based write transactions are split in as many blocks as available accelerators. This process is done on the fly, without incurring additional latency overheads. When using this mode, overlapping periods of memory transactions and data processing occur, since each accelerator starts working as soon as its corresponding data block has been written to its local memory. The resulting burst-based read transaction is composed also on the fly by retrieving data from the accelerators in the same order that was used during the write operation. This mode boosts computing performance using a SIMD-like approach: several copies of a hardware accelerator (same functionality) process different data.

**Figure 5:**
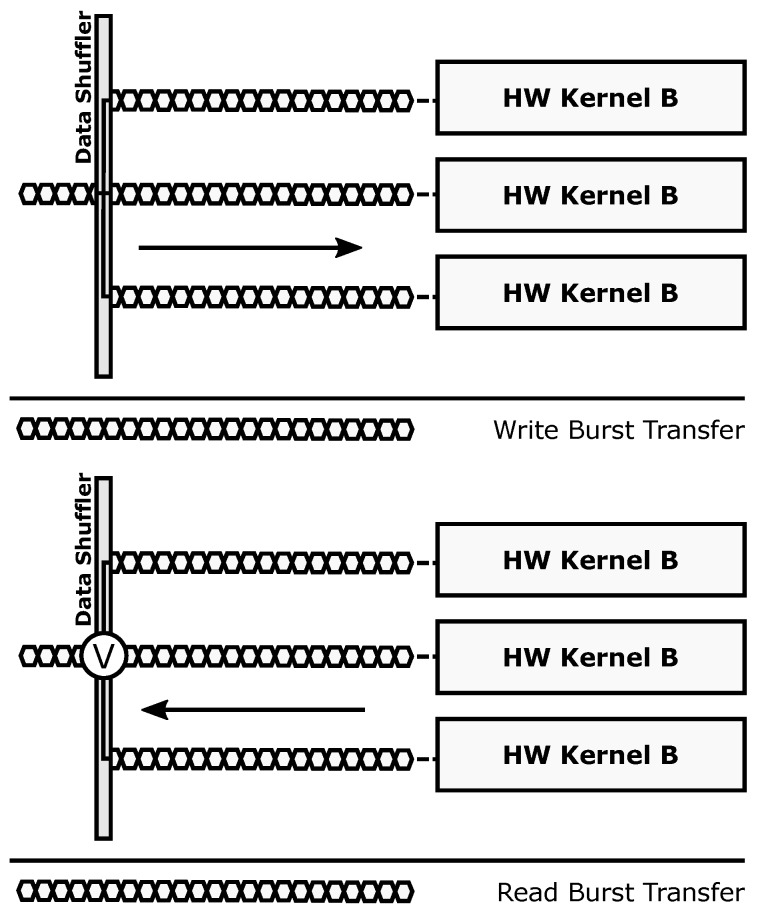
Redundant operation mode.

The redundant transaction mode (Figure 5) is used to enforce fault-tolerant execution, taking advantage of Double or Triple Module Redundancy (DMR and TMR, respectively). Hence, two or three copies of a given accelerator must be present for this mode to be applied. In this mode, burst-based write transactions are issued using a multicast scheme: all the accelerator copies get the same data strictly in parallel. Once the processing is done, a burst-based read transaction is issued, retrieving data also in parallel from all accelerators through a voter unit that decides which result is correct. This unit modifies its behavior according to the required fault-tolerance level, acting as a bypass for compatibility with the parallel mode (i.e., Simplex, no redundancy), a comparator in DMR, and a majority voter in TMR. Notice that the proposed scheme only ensures fault tolerance in the reconfigurable region. The rest of the system, including the Data Shuffler (which can be considered as a single point of failure), is assumed to have been previously hardened, if required, using additional fault tolerance techniques (e.g., configuration memory scrubbers [43]) that are out of the scope of this paper.

**Figure 6:**
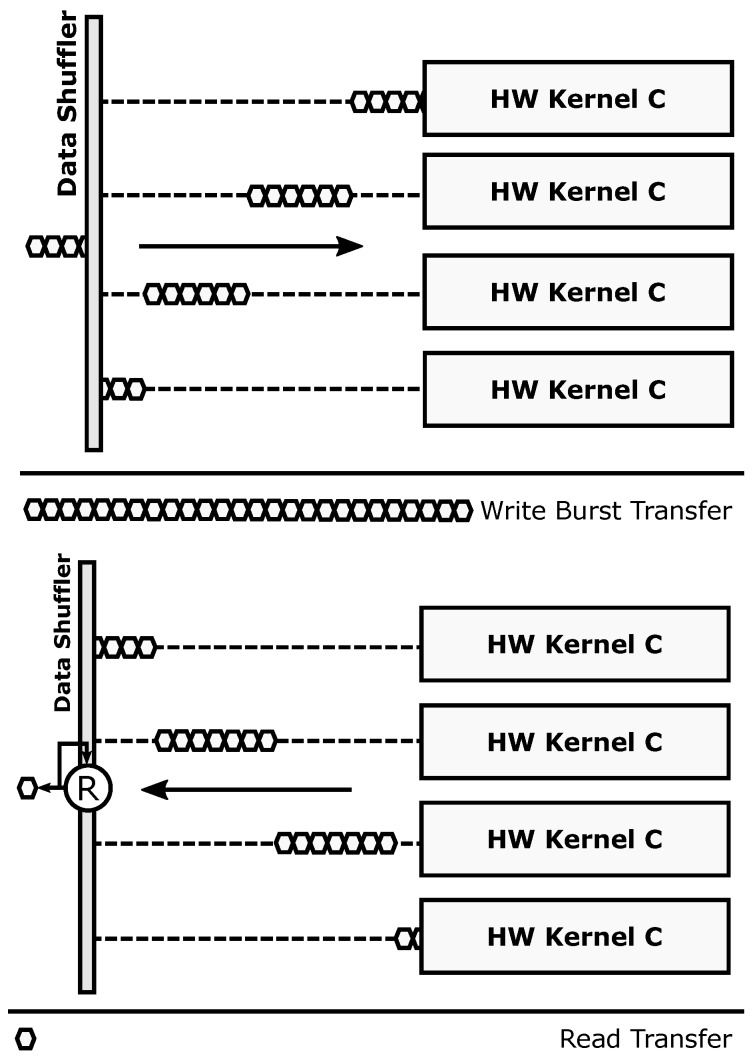
Reduction-oriented operation mode.

The reduction transaction mode (Figure 6) can be thought of as an extension of the parallel transaction mode, where the burst-based read operation is forwarded to an accumulator-based reduction engine before reaching the bus-based communication interface. This mode can be used to enhance computing performance by using a memory transaction not only to move data between the reconfigurable and static domains but also to perform a specific computation (addition, maximum, minimum, etc.) on the fly. An example application where the reduction mode can be used is a distributed dot product, where each accelerator computes a partial result and the reduction unit adds them to obtain the final result with no additional access to the external memory. The proposed accumulator-based approach is inherently efficient: on the one hand, only a small area overhead is introduced (an accumulator, an ALU, and its lightweight control logic); on the other hand, certain computations can be performed without adding latency and memory access overheads other than the ones imposed by data serialization in the bus (which are always present when moving data between memories).

**Figure 7:**
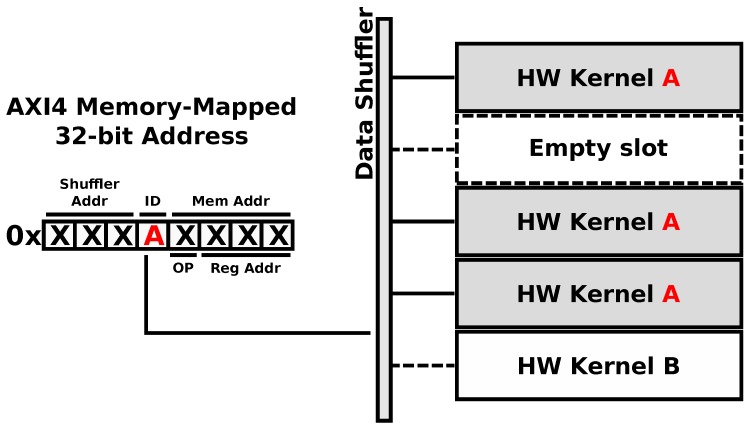
ARTICo^3^ addressing. Accelerator locations are abstracted using an ID that is embedded within the address itself.

The true power of the ARTICo^3^ architecture resides in the method used to access the reconfigurable hardware accelerators, use the different transaction modes and take advantage of the embedded add-on modules (voter unit and reduction engine): the addressing scheme (shown in Figure 7). Taking advantage of the memory-mapped nature of the static region, hardware accelerators are addressed using a unique identifier, which is inserted in the AXI4 destination address for write transactions or in the AXI4 source address for read transactions. Therefore, addressing is made independent of the relative position of a given accelerator in the dynamic region, since it uses virtual sub-address ranges within the global map of the Data Shuffler to address hardware accelerators. Virtual sub-mappings are transparently managed by the framework, making kernel design agnostic from this process. As a consequence of this *smart addressing* scheme, the system can support user-driven task migration without compromising execution integrity in case a reconfigurable slot presents permanent faults (e.g., on-board processing in a satellite might suffer from severe radiation effects). Moreover, the proposed addressing scheme is also used to encode specific operations as part of the transaction addresses. For instance, the destination address in write transactions provides support for multicast commands (e.g., reset all accelerators with a given identifier), whereas the source address in read transactions can be used to encode the operator which is to be used when the reduction mode is enabled.

However, the architecture not only includes an optimized datapath and a smart addressing scheme, but it also features embedded Performance Monitoring Counters (PMCs) to enable self-awareness, and could also be extended by attaching additional sensor interfaces to enable environment-awareness (a common scenario in CPSs, where sensing physical variables is fundamental). There is a number of common PMCs for all implementations, measuring useful performance metrics such as execution times per accelerator, bus utilization (memory bandwidth), or fault tolerance metrics such as the number of errors found per slot (this report comes from the voter unit). In addition, some implementations also feature power consumption self-measurement (provided that the required instrumentation infrastructure is available). As a result, the architecture provides an infrastructure to get relevant information that can be used to make sensible decisions on whether to change the current working point. Nevertheless, closing this feedback loop is out of the scope of this paper, and is assumed to be managed from user applications.

### 3.2. Toolchain

The ARTICo^3^ architecture provides a good starting point for edge computing in CPSs. However, FPGA-based systems, especially when enhanced by DPR, have been traditionally restricted to Academia due to their inherent complexity at both design and run time. To make the design of ARTICo^3^-based systems accessible to wider range of embedded system designers, an automated toolchain has been developed with two main objectives: on the one hand, to encapsulate user-defined hardware accelerators in a standard wrapper and automatically glue them to the rest of the hardware system; and, on the other hand, to generate the required binaries for software and hardware execution. Note that the hardware/software partitioning of the application is a step that needs to be done beforehand, either manually or using an automated tool. The ARTICo^3^ toolchain assumes independent hardware and software descriptions are provided as inputs. The components to enable a transparent use of ARTICo^3^ at run time, which are also required to make the framework accessible, are covered in the next section.

**Figure 8:**
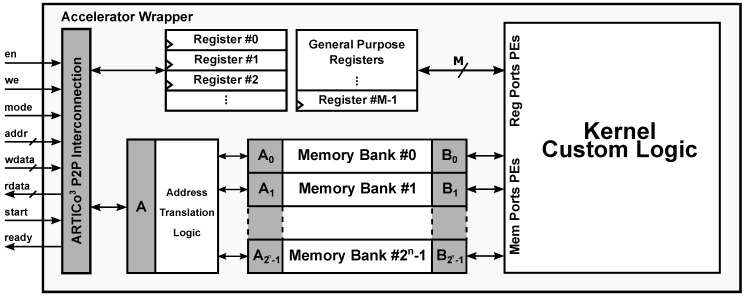
ARTICo^3^ wrapper. The configurable structure (i.e., number of configuration registers or memory banks) is tailored for each functionality that is to be implemented.

The generation of custom hardware accelerators in ARTICo^3^ is very flexible, since developers can choose whether to use low-level HDL descriptions of the algorithms to be accelerated, or use HLS from high-level C/C++ code (the current version of the ARTICo^3^ toolchain only supports Vivado HLS). Either way, the resulting HDL code is then instantiated in a standard wrapper, which provides not only a fixed interface to be directly pluggable in the Data Shuffler, but also a configurable number of memory banks and registers (see Figure 8). The toolchain parses and customizes this wrapper for each kernel that has to be implemented. Hence, the key aspect of the toolchain is that it encapsulates hardware-accelerated functionality within a common wrapper that has a fixed interface with the rest of the architecture, but whose internal structure is specifically tailored for each application. This customization process, together with the generation of DPR-compatible implementations, is performed transparently and without direct user intervention.

The ARTICo^3^ kernel wrapper provides direct connection between user logic and the Data Shuffler using a custom P2P protocol. This protocol relies on enabled read/write memory-mapped operations (enable *en*, write enable *we*, address *addr*, write data *wdata*, and read data *rdata*), a *mode* signal to select the target from memory or registers, two additional control signals to start the accelerator and check whether the processing has finished (*start* and *ready*), and, finally, the *clock* and *reset* signals.

Local memory inside ARTICo^3^ accelerators is specified using two parameters: total size, and number of partitions (or banks) in which it has to be split. This approach increases the potential parallelism that can be exploited by providing as many access ports as necessary, while at the same time keeping all the banks as a uniform memory map for the communication infrastructure (i.e., the DMA engine only sees a continuous memory map for each accelerator, even if more than one bank is present).

Once the accelerators have been generated (i.e., once an HDL description for them is available), the toolchain generates the whole hardware system to be implemented inside the FPGA by using peripheral libraries (either provided by Xilinx or included in the toolchain itself). This process has two different stages: first, a high-level block diagram is generated by instantiating all required modules and connecting them together; second, the block diagram is synthesized, placed and routed, generating FPGA configuration files for both static and dynamic regions (full and partial bitstreams, respectively). In parallel, the toolchain also generates the required software project by combining the user files (where the main application is defined) with the API to access the underlying ARTICo^3^ runtime library, creating a customized Makefile to transparently build the application executable.

**Figure 9:**
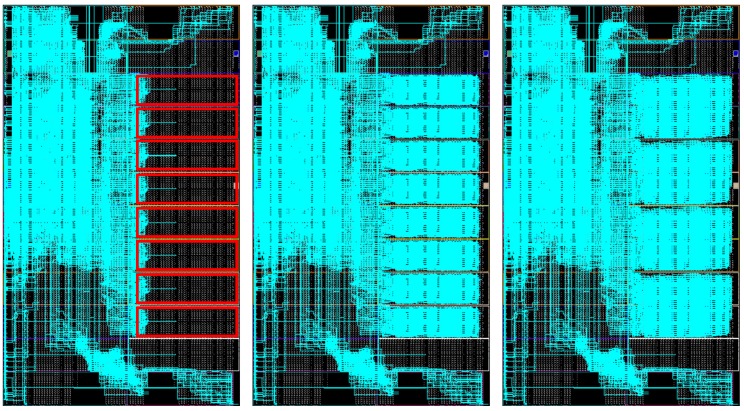
FPGA floorplanning of an ARTICo^3^-based system in the HiReCookie node.

**Figure 10:**
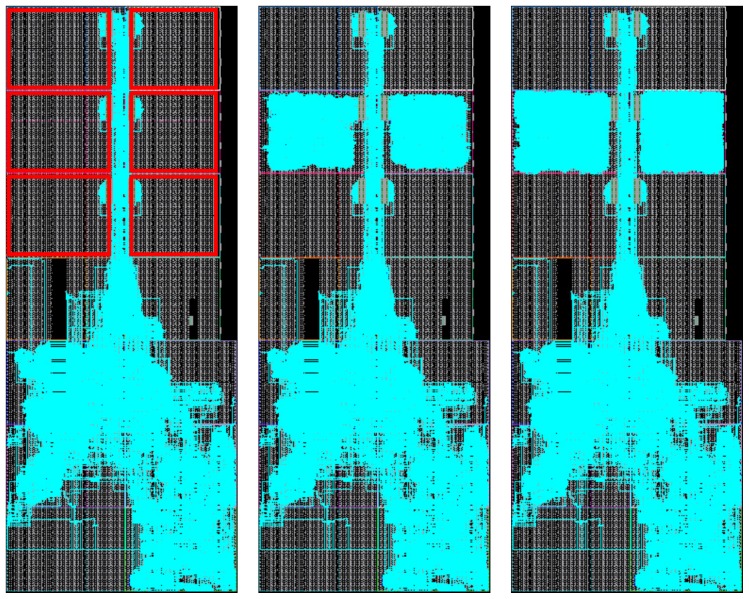
FPGA floorplanning of an ARTICo^3^-based system in the KC705 development board.

**Figure 11:**
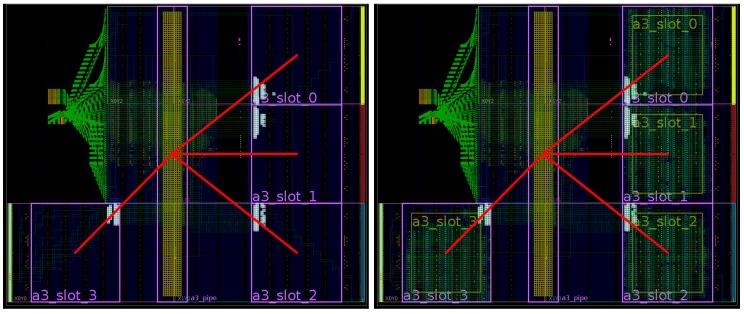
FPGA floorplanning of an ARTICo^3^-based system in a Zynq-based board. Up-to-date design tools (i.e., Vivado) are used for this implementation.

As a result, the toolchain produces output binaries for both hardware and software components without user intervention. This automated methodology greatly increases productivity by reducing development time and error occurrences, especially when compared to legacy design methodologies that were mainly handmade. For example, Figure 9 and Figure 10 show ARTICo^3^-based systems in two different devices that were manually generated and routed using legacy Xilinx tools (ISE), whereas Figure 11 shows the outcome of the toolchain (which uses current Xilinx Vivado tools) for another device. Although they are functionally equivalent, development time went down, on average, from almost a week to a couple of hours (these figures are based on the authors’ experience on designing reconfigurable systems). The main reason for this is that low-level technology and device dependencies are transparently handled by the toolchain, hiding them from the developer, who can now implement DPR-enabled systems with no extra effort.

The ARTICo^3^ toolchain, which is made up of Python and TCL scripts, is highly modular, relying on common constructs that are particularized for each individual implementation (system templates for hardware generation, and application templates for software generation). This is also a key aspect, since it makes adding new devices to the ones supported by the toolchain, or changing the peripherals present for an already existing device, really easy.

### 3.3. Run-Time

As it was already introduced in the previous section, providing designers with the capabilities to transparently generate ARTICo^3^-based reconfigurable systems from high-level algorithmic descriptions is not enough to make the whole framework accessible to embedded system designers with no prior experience in hardware design. In addition, it is also necessary to provide a common interface to link the automatically generated hardware platforms with the user applications, usually written in any programming language (e.g., C/C++), that will use the proposed processing architecture.

To achieve this, a concurrent run-time environment has been also developed. This run-time environment has been implemented as a user-space extension of the application code, relying on a Linux-based OS. Although Linux may not seem the best approach for certain type of embedded systems (e.g., safety-critical applications where specialized OSs are used instead), it shows several advantages. On the one hand, its multitasking capabilities can be exploited to manage different kernels concurrently; and, on the other hand, it is widely used, and developer-friendly.

User applications interact with the ARTICo^3^ architecture and accelerators by means of a runtime library written in C code, which is accessible through a minimal API. The runtime library includes functions to initialize and clean the system, perform DPR to load accelerators, change the working point of the architecture, manage memory buffers between application and accelerators, run kernels with a given workload, etc. Table 2 shows the function calls available in the ARTICo^3^ runtime API.

The ARTICo^3^ run-time environment automates and hides from the user two processes: FPGA reconfiguration and parallel-driven execution management (i.e., workload scheduling over a given number of accelerators using variable-size DMA transfers between external and local memories). However, users are expected to actively and explicitly decide how many accelerators need to be loaded for a given kernel and how to configure them using artico3_load (this function performs DPR transparently). Moreover, users are also in charge of allocating as many shared buffers (between external and local memories) as required by the application/kernels using artico3_alloc. Once everything has been set up, users can call artico3_kernel_execute to start kernel execution. This function takes advantage of the data-independent ARTICo^3^ execution model to automatically sequence all processing rounds (global work) over the available hardware accelerators of a kernel (each of which can process a certain amount of local work). The sequencing process sends data to the accelerators (DMA send transfer), waits for them to finish and then gets the obtained results back (DMA receive transfer) until all processing rounds have finished. The ARTICo^3^ run-time environment creates an independent scheduling/sequencing thread for each kernel that is enqueued for processing. Host code can then continue its execution in parallel to the accelerators until it needs to wait for any of the sequencing threads to finish, which can be done by calling the function artico3_kernel_wait.

### 3.4. Previous Work

The ARTICo^3^ architecture was first introduced in [44]. It originally featured a PLB-based communication infrastructure (for compatibility with legacy Xilinx FPGAs with PowerPC microprocessors), and a reduced set of operation modes that were mutually exclusive. The current implementation features AXI4-based communication infrastructures (for compatibility with modern ARM-based SoCs), and enables configurable and additive transaction modes. At its initial stages, it did not have any associated toolchain or runtime management software.

The first analysis of the solution space exploration capabilities of the ARTICo^3^ architecture was presented in [45], were the data-parallel execution model that enables transparent scalability was also introduced. However, the flow relied on custom ad-hoc bare-metal software libraries and manual hardware implementation techniques that were application-specific and needed to be tailored for each scenario. Currently, the ARTICo^3^ runtime library provides a standard methodology to interface with hardware accelerators from user applications. This library also supports multikernel execution, a feature that was not present in the bare-metal versions of the library. In addition, automatic hardware system generation from kernel descriptions has been provided via the ARTICo^3^ toolchain.

The monitoring infrastructure available in ARTICo^3^ was presented in [46]. This, together with a set of lightweight estimation models, designed to be embedded within the platform itself, enabled the recreation of the solution space in any ARTICo^3^-powered application without actually having to load and change the configuration of the accelerators.

## 4. Experimental Results

This section has two objectives: first, illustrate how the architecture can be actively adapted from user code to explore the solution space, defined by a tradeoff between computing performance, energy consumption and fault tolerance at run-time, for a given application; and, second, compare the proposed solution with a software-based alternative in a common scenario for CPSs.

To this end, four different algorithms have been implemented on the ARTICo^3^ architecture: on the one hand, two image filters based on sliding window algorithms (median and Sobel) and a block ciphering algorithm (AES-256) using HDL descriptions; and, on the other hand, a 32-bit floating point matrix multiplier using C code and HLS. All implementations used are in-house custom designs. These algorithms provide a rich variety of parallelization schemes, making it possible to test the architecture in different scenarios: images can be split in stripes that can be individually processed by different accelerators, block ciphers can work in parallel for certain operation modes (such as the integer counter mode, named CTR in this manuscript), and there are several block-based approaches to make matrix multiplication more efficient. Moreover, each algorithm that has been implemented serves its own purpose. For instance, HDL-based kernels have been chosen to precisely illustrate the way in which the solution space can be explored at run-time, whereas the C-based kernel has been used to prove the feasibility of ARTICo^3^ in a context of energy-efficient edge computing for CPSs.

In addition, three different FPGA-based platforms have been used: a custom high-performance wireless sensor node called HiReCookie that includes a Spartan-6 FPGA (XC6S150-2FGG484) [7], a Xilinx KC705 development board that includes a Kintex-7 FPGA (XC7K325T-2FFG900C), and another custom board that includes a Zynq-7000 SoC (XC7Z020-1CLG484). Manual, legacy implementations have been developed in both the HiReCookie and the KC705 board and use an ad-hoc implementation of the runtime library running as a standalone application in a soft-core microprocessor, whereas the Zynq-based systems have been developed using the ARTICo^3^ toolchain and use the fully-fledged runtime library under Linux (which runs on the ARM cores of the SoPC). Each board has a different number of available slots (due to resource heterogeneity and device size): in the HiReCookie, the reconfigurable region has been divided into 8 slots; in the KC705 board, the partitioning has enabled 6 slots; and, in the Zynq-based board, only 4 slots have been generated after partitioning.

Resource utilization from the ARTICo^3^ infrastructure can be seen in Table 3, where the first column corresponds to the Data Shuffler (both AXI4 interfaces, voter and reduction units, etc.). The second and third columns include only the resources required inside the Data Shuffler to implement the AXI4-Lite and the AXI4-Full interfaces. The fourth and fifth columns show the additional logic, placed outside the Data Shuffler, that needs to be instantiated to generate the ARTICo^3^-based hardware system (i.e., AXI4 Interconnect IP Cores from Xilinx).

Table 4 presents the resource utilization of all the kernel implementations, as well as information regarding their implementation (local memory size, number of memory banks inside each accelerator, number of registers, input source, target board, and number of parallel threads inside each accelerator). An additional column has been added to show the resource utilization of the ARTICo^3^ kernel wrapper with no internal user logic. All these values correspond to one single accelerator (i.e., the contents of one reconfigurable ARTICo^3^ slot).

### 4.1. Solution Space Exploration

As stated in the previous sections, one of the main characteristics of the architecture is that its working point can be changed at run-time from the host application code. These points belong to the so-called solution space of the architecture, which can be represented as a family of curves in a 2D plot (execution time vs. energy consumption for each hardware redundancy level).

**Figure 12:**
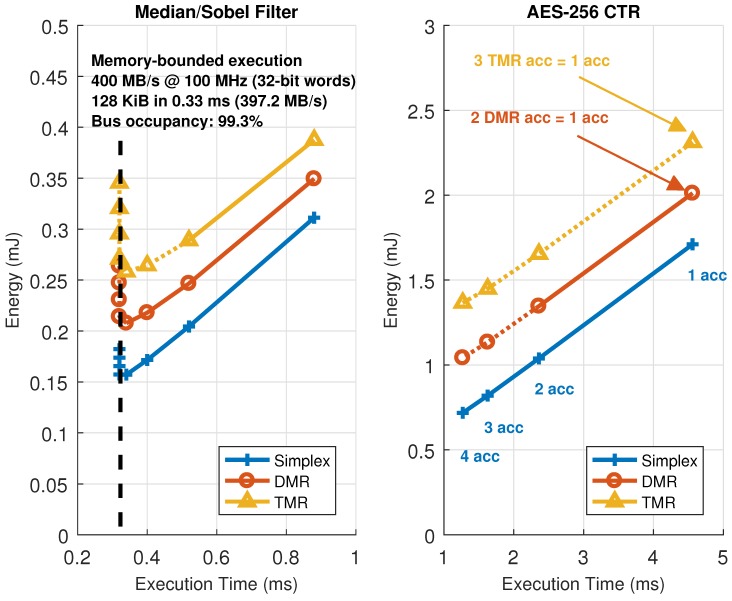
Solution space exploration in the HiReCookie node for memory-bounded (image filters, **left**) and computing-bounded (block cipher, **right**) algorithms.

Figure 12 represents all the possible combinations in number of accelerators and their configuration, which are the parameters that need to be changed to move the working point of the architecture, for the image filtering and encryption accelerators in the HiReCookie node. From these results, it is clear that the image filters show a memory-bounded execution profile, saturating the available bandwidth in the dedicated data bus to almost full occupancy (99.3% of the theoretical maximum). Furthermore, this confirms that, whenever a kernel enters in memory-bounded execution, adding more accelerators to the architecture moves the working point to a worse scenario (more energy is consumed by just having the extra accelerator loaded, but execution performance cannot be increased), thus making working points close to that limit the optimal ones. The block cipher accelerators, on the other hand, always show a computing-bounded execution profile, since no saturation can be observed, and the system can move to better working points by increasing the number of accelerators (less time, and less energy consumption). Two additional comments have to be made regarding these results: the size of the accelerators is not the same (image filters use one slot, whereas the block cipher uses two), and the dotted lines represent solutions that have been estimated using models and kernel profiling using self-measurements [46] because their implementation is not feasible (there are not enough slots to allocate that many accelerators). The estimation process uses run-time measurements during a dummy execution of a given kernel to feed a parametric model and then predict the evolution of the working point of the architecture.

**Figure 13:**
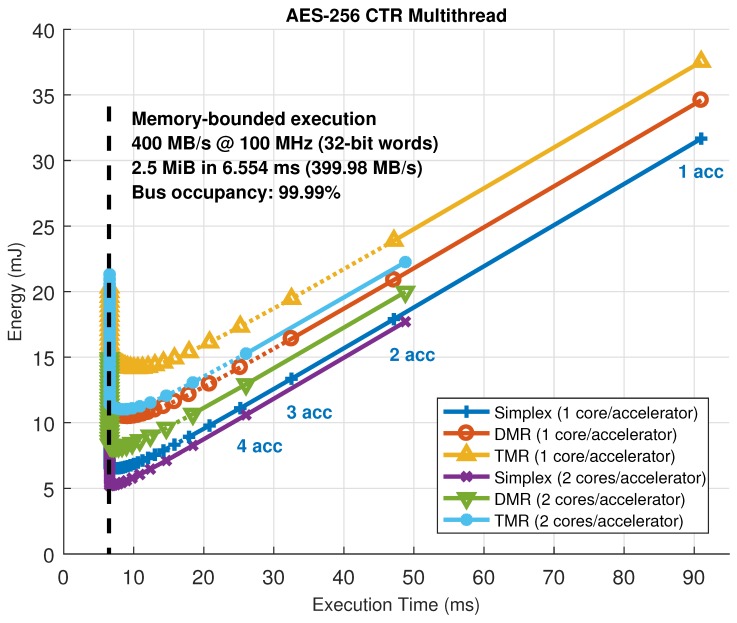
Solution space exploration in the KC705 development board. A computing-bounded algorithm (block cipher) with one or two parallel datapaths per accelerator is used.

On certain occasions, slots are large enough to host accelerators with parallel datapaths inside, thus increasing the amount of parallelism that can be exploited. An example of this situation can be seen in Figure 13, where the same block cipher algorithm has been implemented on the KC705 development board, but using up to six accelerators with one or two internal AES cores operating in CTR mode. In this case, estimations have also been used to predict the maximum achievable performance for this particular setup, rendering a saturation limit of 99.99% in terms of memory bandwidth or bus utilization. Notice that, in this case, one ARTICo^3^ accelerator with two cores inside shows almost the same performance, with less energy consumption, as two ARTICo^3^ accelerators with only one core inside, mainly because it saves up the energy spent by the local memory banks and the associated control logic.

**Figure 14:**
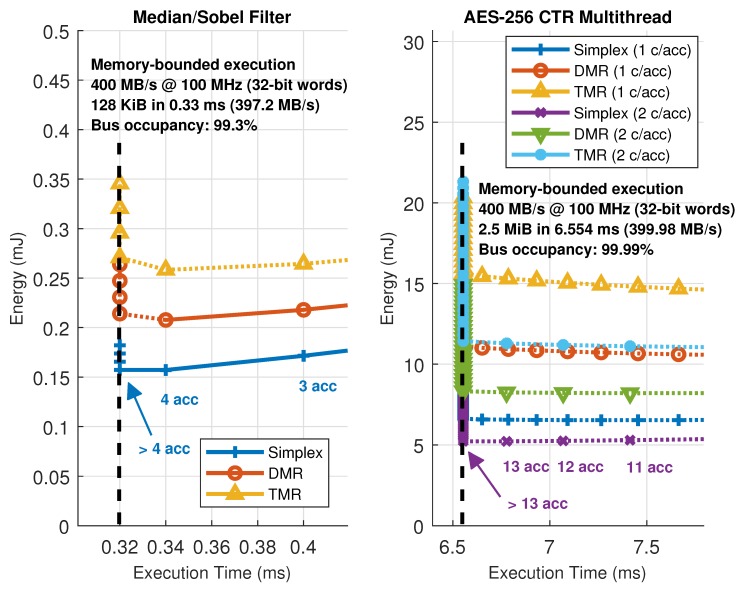
Closeup from the solution space exploration near the bus saturation point (memory-bounded behavior).

Finally, Figure 14 shows a closeup capture of the memory-bounded configurations in each setup. As mentioned above, execution time stalls when reaching a given number of hardware accelerators. However, this situation is not likely to happen for computing-intensive kernels (which in turn are the main target for ARTICo^3^ acceleration). This can be seen in the AES-256 CTR solution space, where the saturation point is far from the maximum feasibility point (i.e., the maximum number of reconfigurable slots), being all memory-bounded points just theoretical estimations obtained with the model.

Given the results presented in this section, it is possible to state that the ARTICo^3^ solution space presents similar trends regardless of the implemented algorithm: almost linear scalability in the computing-bounded region and execution bottleneck when reaching bus saturation (based on bus data width and operating frequency alone) in the memory-bounded region. Hence, it is necessary to analyze the overheads due to the ARTICo^3^ runtime to isolate the infrastructure-related contributions from the algorithm-specific ones.

The foremost contributor to the performance overhead in the ARTICo^3^ infrastructure is memory management. The ARTICo^3^ runtime library provides transparent buffers between user applications and hardware accelerators. These buffers reside in virtual memory to enable fast computation from the software point of view, but they need a shadow counterpart in DMA-allocated (i.e., physical) memory. Whenever a transaction is performed, data must be copied between original and shadow buffers. In addition, setting up the DMA engine to perform a transaction between external and accelerator memories introduces a fixed overhead that does not depend on the number of data to be transferred. This means that, for the same workload to be processed, having more hardware accelerators leads to a better utilization of the DMA engine.

Mean values of memory management overheads for both write and read operations have been measured in a one-accelerator scenario and can be seen in Table 5. Notice that, in a worst-case scenario where the maximum amount of data needs to be transferred, the obtained overhead is less than six clock cycles per 32-bit word (assuming a clock frequency of 100 MHz in the FPGA).

Once the infrastructure-related performance overheads have been characterized, it is possible to also analyze application-specific performance metrics. Table 6 presents a quantitative comparison between a standalone implementation of an AES-256 CTR accelerator with ideal DMA transfers (no idle times) and three different ARTICo^3^ configurations processing the same workload. It is possible to see that, in a one-accelerator scenario, the ARTICo^3^-based solution presents an overhead of 36% over the standalone implementation. However, this performance overhead contrasts with the productivity gain during the design stages (as stated in Section 3.2, development time to build the full reconfigurable system using the automated toolchain went down from a week to a few hours), and it disappears (reaching a gain of up to 33% with four accelerators) due to performance scalability when using the ARTICo^3^ runtime library to adapt the level of parallelism. While it is true that a custom-made system could also use up to four accelerators in parallel, it would require efficient DPR and DMA-enhanced parallel execution management (already available and transparent to the user in ARTICo^3^) to avoid resource underutilization.

Hence, abstracting the analysis from the algorithms, it can be seen that ARTICo^3^ introduces only a small performance overhead for heavily computing-bounded kernels, where DMA transfers are almost negligible when compared with the accelerator processing time. Taking the standalone one-accelerator setup as the reference baseline, it is clear that scalable ARTICo^3^-based computing can improve performance significantly, while at the same time increasing energy efficiency and offering optional fault tolerance.

### 4.2. Energy-Efficient Edge Computing Scenario

For this scenario, a matrix multiplication example has been selected to illustrate the benefits of using ARTICo^3^ as a platform for edge computing in CPSs, since matrix multiplication is a recurrent operation that appears in a wide variety of algorithms used in that context (e.g., neural inference for embedded machine learning in smart sensors).

The reference application multiplies two 512 × 512 32-bit floating point matrices using a hierarchical block-based approach, offloading the computation to one or more ARTICo^3^ accelerators (each accelerator can in turn multiply two 64 × 64 32-bit floating point matrices), and compares the obtained results against a software-based implementation of the naive matrix multiplication algorithm running in one of the ARM Cortex-A9 available in the Processing System of the Zynq device.

**Figure 15:**
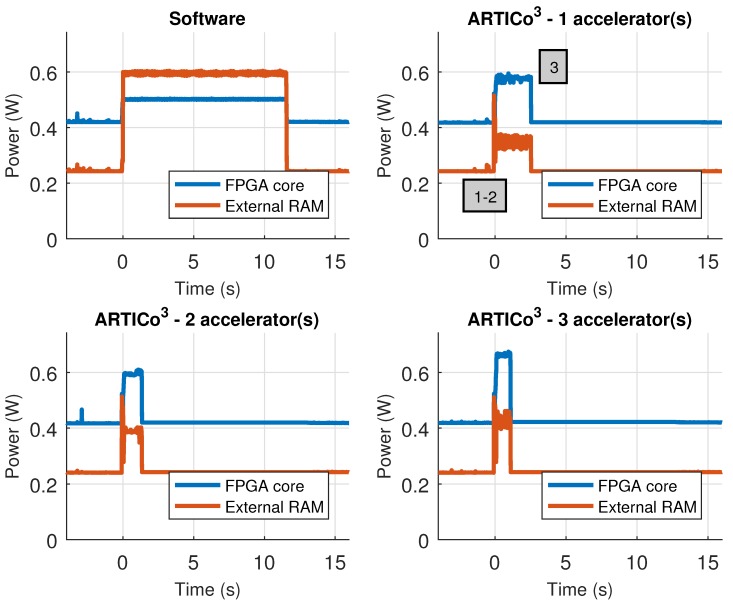
Power consumption profile in the Zynq-based board. Measured power rails are FPGA core and external RAM memory.

The custom Zynq board has support for measuring power consumption using an external ADC connected to two power rails: Zynq core supply and external RAM supply, and hence, the results presented in this section have been measured using the infrastructure available in the board. Table 7 shows the performance metrics (self-measured using the embedded PMCs) when executing the application scenario in software and in ARTICo^3^, changing the number of accelerators used for processing. Even if the time spent reconfiguring the FPGA to load the accelerators is taken into account, the hardware-based implementations are always better than the software-based counterpart. Figure 15, on the other hand, shows the power consumption traces during application execution, also when using software- and hardware-based processing with a variable number of ARTICo^3^ accelerators. For the hardware solutions, the measured profile has an initial stage where the FPGA is fully reconfigured to load the bitstream with the static part (i.e., full reconfiguration), a second stage where DPR is used to load as many accelerators as required (i.e., partial reconfiguration, where only the slots are reconfigured), and a third one in which the matrix multiplication is done using ARTICo^3^. Again, note that, even if reconfiguration times are taken into account, the hardware-based solution is still better than the software-based one.

**Figure 16:**
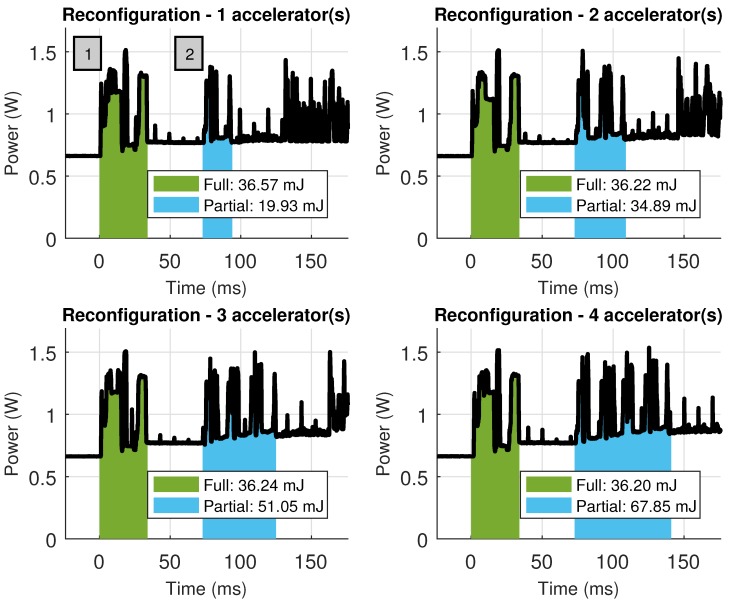
Energy consumption in the Zynq-based board during reconfiguration (either full or partial) when changing the number of accelerators.

Due to the large difference in elapsed times between hardware and software, it is hard to appreciate the reconfiguration stages in detail. Notice that this is the desired scenario, with negligible reconfiguration times when compared with processing times. Nevertheless, Figure 16 shows a close up of both reconfiguration processes, where it is possible to see not only the time spent in these processes but also the energy consumed. Since reconfiguration is performed using the PCAP (Processor Configuration Access Port) available in Zynq devices, which is a DMA-powered reconfiguration engine, loading large bitstreams is less time consuming than loading smaller, partial bitstreams into the FPGA (one reconfiguration command and its associated DMA transaction are issued per partial bitstream). This can be seen in the scenario where four accelerators are loaded (bottom right), spending also more energy during the process than the energy spent during full FPGA reconfiguration.

**Figure 17:**
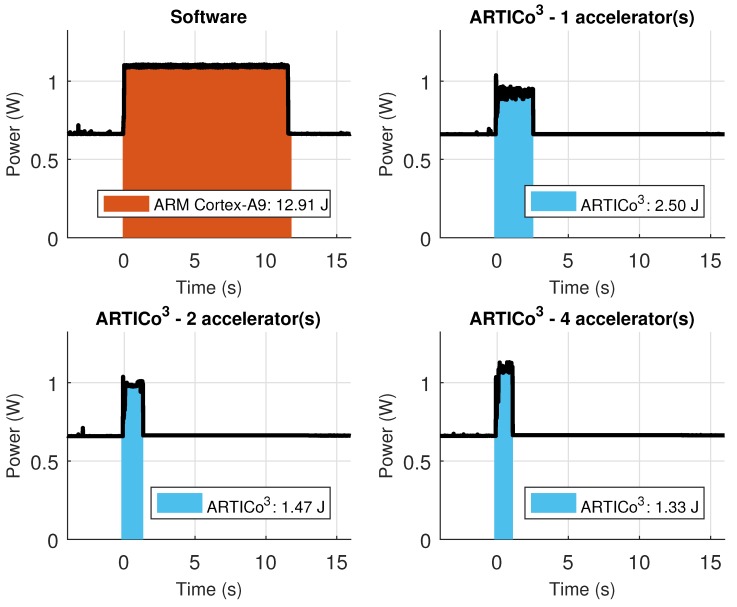
Energy consumption in the Zynq-based board during both software- and ARTICo^3^-based processing when changing the number of accelerators.

However, having higher power consumption values is not bad per se, since it can then lead to faster processing times and, therefore, less energy consumption during the hardware-based processing stages (provided that the algorithm or application is highly intensive in terms of computing requirements). This scenario can be seen in Figure 17, where the energy spent by the example application is measured in four different scenarios (only software, and ARTICo^3^ with one, two or four accelerators). Notice that the measured values for hardware-based computing also include the energy spent during full and partial reconfiguration, proving that sometimes it is beneficial to use more accelerators than originally intended, provided that the working point has been set close to the computing-bounded region of the solution space. Under these circumstances, it is possible to achieve a reduction of 9.5 times in terms of energy, achieving also a speed up of 13.05 times in terms of execution performance.

## 5. Conclusions and Future Work

In this paper, a framework to develop high-performance embedded systems for edge computing in CPSs has been presented. The proposed approach provides a hardware-based processing architecture called ARTICo^3^ and the required tools, at both design and run time, to ease the development of user defined applications. This is achieved by hiding from the end user low-level technology dependencies (and limitations) such as hardware design and DPR (usually considered out of reach for many system developers), making FPGAs more accessible to the general public.

The architecture provides support for establishing tradeoffs between computing performance, energy consumption and fault tolerance at run time, enabling designers to dynamically explore the solution space for any given application. This analysis of all possible working points (even those corresponding to configurations that are not feasible) is possible thanks to self-awareness techniques (via embedded monitors and estimation models), as it has been demonstrated through experimental results.

Moreover, the benefits of using the framework to generate edge computing systems have been evaluated using a computational-intensive algorithm as the reference application scenario, rendering significantly better results in terms of both computing performance and energy consumption when compared with equivalent software-based platforms (13× more computing performance, 9.5× less energy consumption). These are key aspects when moving computation close to where information is generated and, thus, ARTICo^3^ appears as a solid and feasible alternative to mainstream high-performance embedded computing platforms for data-parallel algorithm acceleration.

The use of Linux has enabled a proof-of-concept implementation and validation, mainly due to simplicity in the integration process. Nevertheless, the excessive and non-uniform overhead generated by the OS itself affects basic features of the framework such as solution space exploration. In this regard, the use of a Real-Time Operating System (RTOS) is envisioned as a future line of work, since it will remove the unpredictable behavior and will ensure determinism during run time.

Processing performance, energy consumption, and fault tolerance are key aspects in CPS end devices, and experimental results show that ARTICo^3^-based Edge Computing can be very suitable for them. Extending this idea by using small-sized ARTICo^3^-based computing clusters to not only perform Edge but also Energy-Aware and Fault-Tolerant Fog Computing is also envisioned as another future line of work. This approach is expected to significantly increase the set of applications that can benefit from the ARTICo^3^ framework, while still keeping processing close to data sources.

## Figures and Tables

**Table 1:** Design tools comparison.

Tool	Kernel Specification Entry Point	DPR Support	Parallelism Exploited	Support for Dynamic Adaptation at Runtime
**RecoBus-Builder**	VHDL/Verilog	Accelerator	Not Explicit	No
**GoAhead**	VHDL/Verilog	Accelerator	Not Explicit	No
**SDAccel**	C/C++, OpenCL	Accelerator Group	Task, Data	No
**SDSoC**	C/C++, OpenCL	No	Task	No
**ARTICo^3^**	VHDL/Verilog, C/C++	Accelerator	Task, Data	Yes

**Table 2:** ARTICo^3^ Runtime API.

Group	Function	Description
**General**	artico3_init	Initializes the runtime and loads the static system
	artico3_exit	Releases the runtime and cleans environment
**Slot**	artico3_load	Loads a kernel in a slot and sets its configuration
	artico3_unload	Removes any previous slot configuration
**Memory**	artico3_alloc	Allocates a DMA-capable memory buffer for a kernel port
	artico3_free	Frees the memory buffer associated to a kernel port
**Kernel**	artico3_kernel_create	Registers a kernel in the runtime (gets an ID)
	artico3_kernel_release	Removes a kernel from the runtime (frees the ID)
	artico3_kernel_execute	Executes a kernel asynchronously (delegate thread)
	artico3_kernel_wait	Waits for kernel completion
	artico3_kernel_reset	Reset all accelerators of a given kernel
	artico3_kernel_wcfg	Writes data to kernel registers
	artico3_kernel_rcfg	Reads data from kernel registers
**Monitor**	artico3_hw_get_pmc_<name>	Access the specific PMC and gets its current value

**Table 3:** Resource utilization: ARTICo^3^ infrastructure (Zynq-7000).

	ARTICo^3^			Static System	
Component	Data Shuffler	Data Shuffler AXI4-Lite Slave IF	Data Shuffler AXI4-Full Slave IF	Control Bus AXI4-Lite	Data Bus AXI4-Full
**LUTs**	4142	1669	520	449	0
**FFs**	2365	409	256	598	0

**Table 4:** Resource utilization: ARTICo^3^ kernels.

	ARTICo^3^ Wrapper	Median Filter	Sobel Filter	
**Info**	64 KiB, 4 banks	16 KiB, 2 banks	16 KiB, 2 banks	
	8 registers	8 registers	8 registers	
	-	VHDL	VHDL	
	-	HiReCookie	HiReCookie	
	-	1 thread	1 thread	
**LUTs**	959	1136	1035	
**FFs**	545	1123	819	
**DSPs**	0	0	0	
**BRAMs**	16	4	4	
	**AES-256 CTR**			**Matrix Multiplier**
**Info**	32 KiB, 2 banks	64 KiB, 2 banks	64 KiB, 4 banks	48 KiB, 3 banks
	8 registers	8 registers	8 registers	0 registers
	VHDL	VHDL	VHDL	C + HLS
	HiReCookie	KC705	KC705	Zynq Custom
	1 thread	1 thread	2 threads	1 thread
**LUTs**	3710	3721	7005	3292
**FFs**	3479	3484	6329	2786
**DSPs**	0	0	0	20
**BRAMs**	9	17	18	16

**Table 5:** Memory management overheads.

Operation	memcpy Time (ns/B)	DMA Time (ns/B)	Total Overhead (ns/B)
**Write**	8.85	5.43	14.28
**Read**	7.57	5.98	13.55

**Table 6:** Performance evaluation for AES-256 CTR: custom design (theoretical bound) vs. ARTICo^3^ (evaluation based on processing 512 KiB @ 100 MHz).

Implementation	Processing (ms)	Data Transfer (ms)	Total Time (ms)	Overhead
**Monolithic + Ideal DMA**	33.75	2.62	36.37	–
**ARTICo^3^ (1 Accelerator)**	33.75	15.73	49.48	+36%
**ARTICo^3^ (2 Accelerators)**	16.88	15.73	32.6	−10%
**ARTICo^3^ (4 Accelerators)**	8.44	15.73	24.17	−33%

**Table 7:** Matrix multiplication: performance metrics.

Implementation	Accelerators	Reconfiguration Time (ms)	Processing Time (s)	Speedup
**Software**	-	-	11.51	1
**ARTICo^3^**	1	59.61	1.76	6.54
	2	74.47	1.12	9.74
	3	106.72	0.97	11.88
	4	142.21	0.89	13.05
